# Cavitation-Enhanced Drug Delivery and Immunotherapy

**DOI:** 10.3390/pharmaceutics15092207

**Published:** 2023-08-26

**Authors:** Brandon Helfield, Shashank Sirsi, James Kwan, Michael Gray

**Affiliations:** 1 Department of Physics, Concordia University, Montreal, QC H3G 1M8, Canada; brandon.helfield@concordia.ca; 2 Department of Biology, Concordia University, Montreal, QC H3G 1M8, Canada; 3 Erik Jonsson School of Engineering and Computer Science, The University of Texas at Dallas, Richardson, TX 75080, USA; shashank.sirsi@utdallas.edu; 4 Department of Engineering Science, University of Oxford, Oxford OX1 3PJ, UK; james.kwan@eng.ox.ac.uk; 5 Biomedical Ultrasonics, Biotherapies and Biopharmaceuticals Laboratory, University of Oxford, Oxford OX3 7LD, UK

Welcome to this special issue on Cavitation-Enhanced Drug Delivery and Immunotherapy—a rapidly evolving area that has been buoyed in recent years by the development of methods harnessing the activity of ultrasound-stimulated bubbles known as cavitation. When properly controlled, cavitation can help overcome physical barriers to drug delivery whilst providing readily measurable information for timely quantitative feedback and treatment guidance. Microbubble-assisted therapies have demonstrated impressive advancements in clinical trials and pre-clinical areas, including applications in neurology, oncology, cardiology and beyond.

In this special issue we have eight original research contributions highlighting the breadth of targets for cavitation-enhanced therapies, including Imran et al. [1]. (pancreas), Lacerda et al. [2] (head and neck cancer), and Ahmed et al. [3] (brain), who all discuss progress in fighting long-challenging pathologies. Imran et al. demonstrated substantially enhanced drug uptake in a porcine pancreatic tumor model using the SonoTran system, which is currently in a clinical trial for targeted chemotherapy to liver tumors. Lacerda et al. explored the treatment parameter space for a head and neck cancer combination therapy, showing the relative importance of O_2_ microbubbles (MBs), mitochondrial respiration inhibitors, and radiation dose rate. In a murine glioma model study, Ahmed et al. demonstrated that an ultrasound mediated blood-brain barrier opening treatment following administration of anti-PD-L1 markedly improved overall survival in comparison to giving anti-PD-L1 alone. 

Some newer targets and applications are also featured in the special issue. LuTheryn [4] et al. describe the first use of cationic MBs for targeted treatment of biofilms, which are notoriously drug-resistant and typically are negatively charged. Cationic MBs were also used by He et al. [5] to safely deliver angiogenic microRNA with an eye toward treatment of cardiovascular disease. Kerneis et al. [6] demonstrated the safety of sonoporation to facilitate inner ear drug delivery. Benton et al. [7] showed the importance of perfluoropentane droplet concentration in the therapeutic context of cardiovascular reperfusion injury. Finally, Martinez et al. [8] present cavitation dose relationships to microbubble size distributions and exposure conditions, and they suggest that gas volume fraction may be used as a unifying factor describing bubble behavior across a range of sizes and concentrations. 

This collection also features four reviews, beginning with Navarro-Becerra and Borden [9] writing on the design and application of targeted microbubbles, challenges in their clinical implementation, and opportunities for protocol optimization and reporting standardization. Chapla et al. [10] reviewed design strategies for microbubble-nanoparticle complexes, highlighting the wide variety of available constructs, while noting the need for near term large animal safety and efficacy studies on the path to clinical evaluation. Honari and Sirsi [11] also discuss ultrasound-sensitive particles, with notably clear descriptions of mechanisms by which mechanical and biological barriers to drug delivery may be overcome. The collection concludes with a review by Maardalen et al. [12] highlighting the potential role of cavitation in modulating anti-tumor immunity and identifying critical areas for further study such as the immunological effects resulting from cavitation activity ranging from gentle oscillation to violent collapse.

Taken together, this collection highlights the exciting progress and prospects for cavitation-mediated therapies, and we hope the information herein both informs and stimulates the readers to make the next important steps to understand and clinically translate techniques for cavitation-enhanced drug delivery and immunotherapy.
